# Gallbladder cancer with tumor thrombus in the portal vein: A case report

**DOI:** 10.1097/MD.0000000000010271

**Published:** 2018-04-20

**Authors:** Xiao-Zhen Zhang, Jian-Jun Tu, Wei Chen, Tao Ma, Xue-Li Bai, Ting-Bo Liang

**Affiliations:** a Department of Hepatobiliary and Pancreatic Surgery, the Second Affiliated Hospital, Zhejiang University School of Medicine, Hangzhou; b Department of Surgery, Kaihua People's Hospital, Quzhou, China.

**Keywords:** gallbladder cancer, portal vein, tumor thrombus

## Abstract

**Rationale:**

Gallbladder cancer (GBC) is a highly fatal malignancy. Due to its invasiveness and delayed diagnosis, many GBC patients are diagnosed with synchronous liver and hepatoduodenal ligament involvement. In our case, we report a gallbladder cancer with portal vein thrombus.

**Patient concerns:**

A 60-year-old woman presented with persistent upper abdominal dull pain for 2 months.

**Diagnoses:**

Ultrasound examination showed gallbladder carcinoma invading liver segment IV, and a tumor thrombus in the left and right main portal trunk. Ultrasonography and contrast-enhanced magnetic resonance imaging (MRI) showed gallbladder carcinoma with invasion of adjacent liver, and tumor thrombus in the right branch of the portal vein and intrahepatic bile duct. Abdominal computed tomography angiography (CTA) revealed no hepatic artery invasion.

**Interventions:**

We made a decision to perform extended right lobectomy. Twenty-six days later, the patient underwent intravenous infusion port implantation for S-1 plus oxaliplatin (SOX) therapy.

**Outcomes:**

After treatment, the patient has been doing very well and no recurrence has been found for 5 months.

**Lessons:**

The patient with gallbladder cancer and tumor thrombus in the portal vein described in this report provides a reminder for surgeons of the importance of early diagnosis, and adequate surgical and adjuvant treatment. Multi-disciplinary treatment is significantly beneficial for the overall survival of patients with advanced GBC.

## 1 Introduction

Gallbladder cancer (GBC) is a highly fatal disease, has a low response to treatment, and a poor overall prognosis, due to detection at a late stage. The 5-year survival of GBC patients is reported to be between 0% and 10% in most reported series.^[1,2]^ It is the most common malignant tumor of the bile duct system and the fifth most common malignant neoplasm of the digestive tract.^[1–3]^ Moreover, although early GBC patients do not have specific symptoms, such as right hypochondrium pain, weight loss, and anorexia, advanced patients may present with a palpable mass, hard nodular liver, hepatomegaly, ascites, gastrointestinal, bleeding, duodenal obstruction, and jaundice which is common and an indicator of poor prognosis.^[1,4]^

Portal vein thrombosis (PVT) is a frequent complication of hepatocellular carcinoma (HCC).^[5]^ The portal vein is very rarely involved by gallbladder tumors or thrombosis due to pressure effects.^[6]^ Here, we report a patient who was diagnosed with GBC and tumor thrombus in the right branch of the portal vein.

## 2 Case report

**Figure 1:**
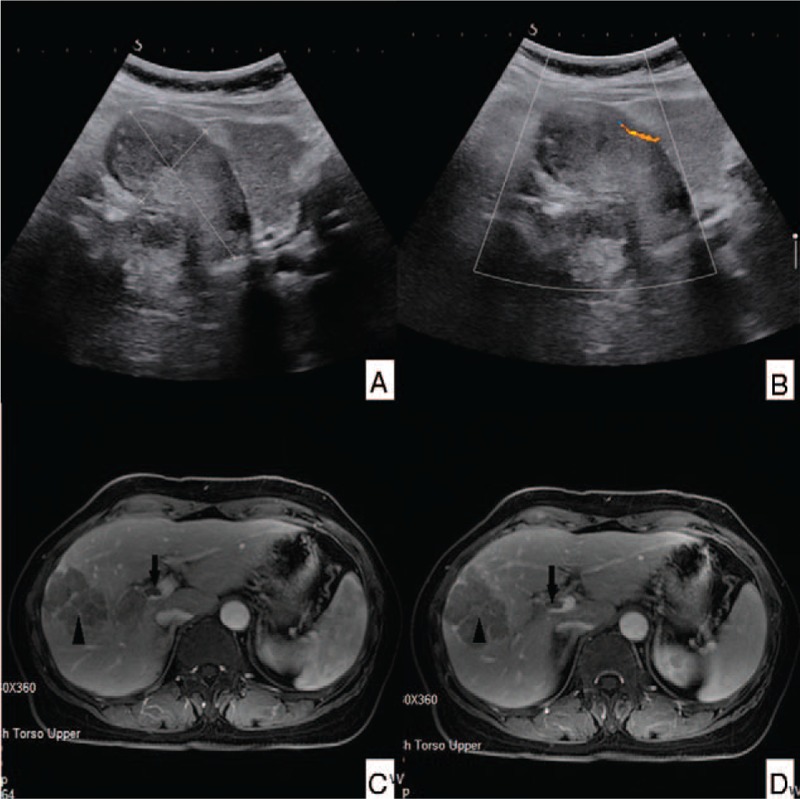
Abdominal ultrasonography (US) (A and B) and contrast-enhanced magnetic resonance imaging (MRI) (C and D). A and B: abdominal US shows gallbladder carcinoma with invasion of adjacent liver, and tumor thrombus in the main portal vein. C and D: Contrast-enhanced MRI shows tumors in liver segment IV and the gallbladder, and tumor thrombus in the right branch of the portal vein.

**Figure 2:**
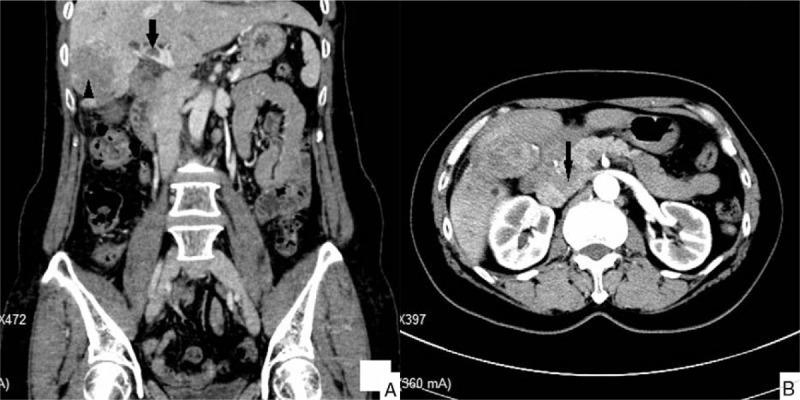
Abdominal computed tomography angiography (CTA) (in coronal scanning image) (A) and contrast-enhanced computed tomography (CT) (in venous phase) (B). A large mass in the right anterior inferior segment to the left medial segment of the liver (arrowhead) and thrombus (arrow) in the main portal vein.

**Figure 3:**
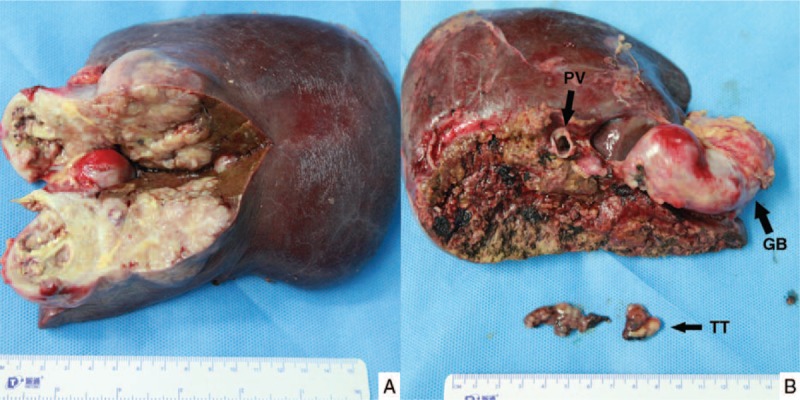
Appearance of the resected specimen. A: The cut surface of the resected specimen. B: Tumor thrombus from the right main portal vein. GB = gallbladder, PV = portal vein, TT = tumor thrombus.

**Figure 4:**
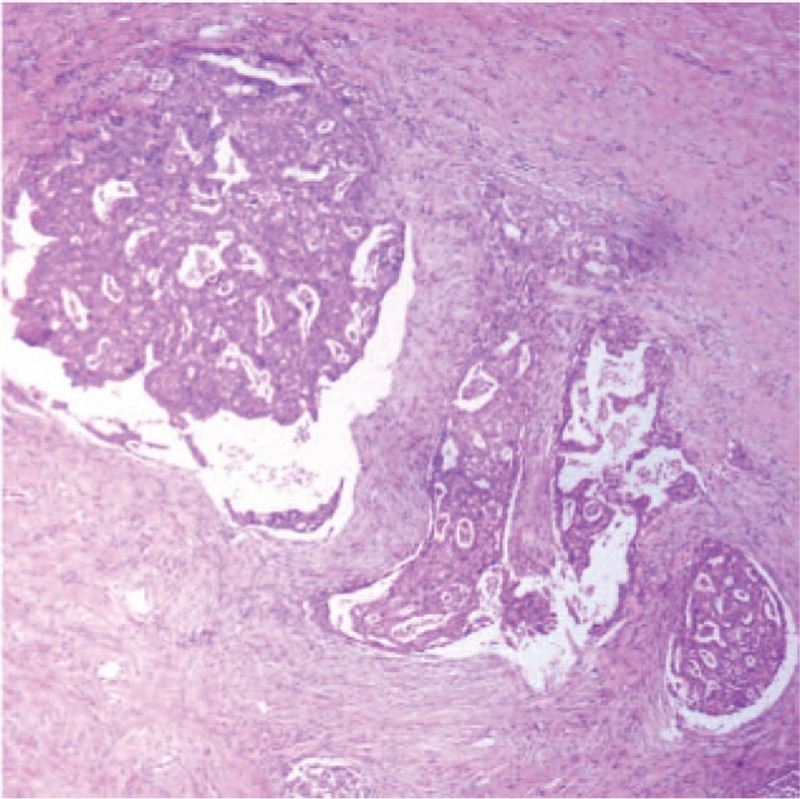
Histological examination. The tumor was a moderately-differentiated adenocarcinoma with invasion of the gallbladder layer and extrahepatic connective tissues, with tumor thrombus in the portal vein.

A 60-year-old woman presented with persistent upper abdominal dull pain for 2 months. Physical examination was unremarkable. She had been diagnosed with asymptomatic cholecystolithiasis without previous treatment. Laboratory tests revealed a slight elevation of carbohydrate antigen 19–9 (CA19–9) (53.5 U/mL; normal, <37 U/mL), and a decreased level of serum total protein (65.0 ng/L; normal, 35.0–52 ng/L). Ultrasound examination showed gallbladder carcinoma invading liver segment IV, and a tumor thrombus in the left and right main portal trunk. Ultrasonography and contrast-enhanced magnetic resonance imaging (MRI) showed gallbladder carcinoma with invasion of adjacent liver, and tumor thrombus in the right branch of the portal vein and intrahepatic bile duct. There was no thrombus in the extrahepatic bile duct (Fig. 1). Abdominal computed tomography angiography (CTA) revealed no hepatic artery invasion (Fig. 2). Blood examination, liver function, and chest CT were normal. Based on the examination results and multi-disciplinary team discussions, we diagnosed adenocarcinoma that had originated in the gallbladder with liver invasion, and had then grown into the right portal vein trunk. According to the preoperative diagnosis and indocyanine green retention at 15 min (ICG15) (7.5%; normal, <10%), the patient underwent peritoneal adhesiolysis, cholecystectomy, choledochotomy, tissue biopsy, extended right lobectomy and lymph node dissection on September 13, 2017. During surgery, no ascites or peritoneal dissemination was observed. Right segment and part of the segment IV were resected and the regional lymph nodes around the hilum were also dissected. Macroscopically, the tumor which was measured 8 × 4 × 3 cm was located in the gallbladder, with another tumor in the right lobe of the liver close to the gallbladder bed. The size of the liver metastasis was 8 × 6 cm. Lymph node metastasis was not observed. In addition, tumor thrombus had grown into the right portal trunk (Fig. 3). Histological examination showed that the tumor was a moderately-differentiated adenocarcinoma with invasion of the gallbladder layer and extrahepatic connective tissues, with tumor thrombus in the portal vein (Fig. 4).

The patient was admitted to our hospital 26 days later for re-examination and adjuvant therapies. Serum CA19–9 was normal (4.7 U/mL; normal, <37 U/mL). Other laboratory tests and imaging examinations were also with in the normal range. The patient underwent intravenous infusion port implantation for S-1 plus oxaliplatin (SOX) therapy. In latest re-examination (131 days later), patient was doing very well, and SOX therapy was continued to performed. According to American Joint Committee on Cancer 8^th^ edition Tumor Node Metastasis staging, the disease stage was defined as T4N0M0, IVA.^[7]^

## 3 Discussion

GBC is a common and aggressive gastrointestinal neoplasm.^[5]^ The poor prognosis of GBC is due to the nonspecific symptoms and signs in the early stage, leading to difficulty in accurate diagnosis.^[1]^ GBC metastasis can result in local invasion, lymphatic metastasis, hematogenous metastasis, and implantation metastasis. In addition, the classification of lymphatic pathways from the gallbladder include: the cholecysto-retropancreatic, the cholecysto-celiac, the cholecysto-mesenteric pathway, and the hilar route that ascends directly toward the hepatic hilum.^[8]^ Jaundice usually occurs due to obstruction of the common bile duct when the hilum is invaded. However, in our patient with GBC and tumor thrombus in the portal vein, the level of total bilirubin was normal. The reason for this is that: gallbladder cancer cells can grow into the portal vein trunk via the invasion of the liver, and not the hepatohilar bile duct. HCC with portal vein tumor thrombosis and gallbladder carcinoma with tumor thrombus in the bile duct/superior vena cava are frequently reported^[9–12,4]^; however, GBC with liver metastatic lesion and thrombosis in the right branch of the portal vein is rare. To the best of our knowledge, only 2 cases have been reported in the literature.^[13,14]^

For patients with GBC complicated with PVT, preoperative diagnosis is vital to improve prognosis, especially in patients with no specific symptoms. Abdominal ultrasound is usually the first imaging method used to diagnose GBC. However, the sensitivity of ultrasound in diagnosing GC is only about 44%.^[15]^ Serum tumor biomarkers such as CA19–9 should be determined, as the presence of elevated of CA19–9 provides strong evidence of GBC. When GBC with tumor thrombus in the portal vein is suspected, abdominal computed tomography (CT) and contrast-enhanced MRI, which are important in the evaluation of GBC, should be performed. However, MRI is not sensitive enough to detect lymph node metastasis (57%).^[16]^ In order to improve the sensitivity and specificity for vascular invasion, CTA, and magnetic resonance angiography (MRA) are recommended. In this case, CT and contrast-enhanced MRI correctly displayed GBC with tumor thrombus in the right branch of the portal vein, while ultrasound failed. Although endoscopic ultrasound (EUS) is a more accurate imaging examination for diagnosis of the GBC than conventional abdominal US, some studies have revealed that CT, MRI combined with CTA, or MRA were more reliable than EUS.^[15,16]^ Thus in our case, EUS was not performed.

Surgery is considered the most effective treatment for GBC. The 5-year overall survival of patients with GBC who underwent R0 curative resection was reported to range from 21% to 69%, and 0% for patients who did not undergo R0 resection. Thus, achieving a complete resection and obtaining negative margins (R0 resection) is important in the surgical management of GBC.^[17,18]^ In our case, an extended right hepatic lobectomy was performed, due to GBC with invasion of the liver and right main portal vein thrombosis. In the past, the role of adjuvant therapy in the treatment of GBC was unclear. However, since 2004, 4 retrospective reports have shown that adjuvant therapy for advanced GBC can significantly improve overall survival.^[19–22]^ Adjuvant chemoradiation therapy is recommended for lymph node-positive GBC or T2/T3 GBC with unknown lymph node status after surgical resection.^[23,24]^ In addition, chemotherapy with or without radiotherapy is administered according to the patient's general condition. Chemoradiation therapy is administered on the premise that the general condition of patients with T2–3NxM0 stage is good. In patients with more advanced stages, radiotherapy cannot be administered. In our case, adjuvant therapy was, S-1 plus oxaliplatin (SOX) therapy which is used as neoadjuvant chemotherapy for gastric cancer and colorectal cancer.^[19,25]^ Radiotherapy was not performed in our patient, who was at T4N0M0, IVA stage.

## 4 Conclusions

According to the above discussions, we can safely infer the following disease course in our case: gallbladder cancer cells migrated and invaded to the gallbladder bed in the liver, resulting in tumor growth in the liver and the cancer cells then grew in the lumen of the portal vein. Early diagnosis of GBC is difficult due to nonspecific symptoms; however, appropriate surgery and adjuvant therapy can improve survival. Hence, this case provides a reminder for surgeons of the importance of early diagnosis, adequate surgical and adjuvant therapy in patients with GBC, especially tumor biomarker such as serum CA19–9 that play an important role in early diagnosis should be paid more attention.

## Author contributions

**Conceptualization:** Xueli Bai.

**Data curation:** Wei Chen.

**Formal analysis:** Wei Chen.

**Supervision:** Xueli Bai, Tingbo Liang.

**Visualization:** Tingbo Liang.

**Writing – original draft:** Xiaozhen Zhang, Jianjun Tu, Tao Ma.

**Writing – review & editing:** Xiaozhen Zhang, Xueli Bai.
